# Social support and fear-inhibition: an examination of underlying neural mechanisms

**DOI:** 10.1093/scan/nsae002

**Published:** 2024-01-12

**Authors:** E.A Hornstein, C J Leschak, M H Parrish, K E Byrne-Haltom, M S Fanselow, M G Craske, N I Eisenberger

**Affiliations:** Department of Psychology, University of California, Los Angeles, CA 90095, USA; Department of Psychology, University of California, Los Angeles, CA 90095, USA; Department of Psychology, University of California, Los Angeles, CA 90095, USA; Department of Psychology, University of California, Los Angeles, CA 90095, USA; Department of Psychology, University of California, Los Angeles, CA 90095, USA; Department of Psychiatry and Biobehavioral Sciences, University of California, Los Angeles, CA 90095, USA; Department of Psychology, University of California, Los Angeles, CA 90095, USA; Department of Psychiatry and Biobehavioral Sciences, University of California, Los Angeles, CA 90095, USA; Department of Psychology, University of California, Los Angeles, CA 90095, USA

**Keywords:** social support, fear-inhibition, retardation-of-acquisition, prepared fear suppressors, ventromedial prefrontal cortex

## Abstract

Recent work has demonstrated that reminders of those we are closest to have a unique combination of effects on fear learning and represent a new category of fear inhibitors, termed prepared fear suppressors. Notably, social-support-figure images have been shown to resist becoming associated with fear, suppress conditional-fear-responding and lead to long-term fear reduction. Due to the novelty of this category, understanding the underlying neural mechanisms that support these unique abilities of social-support-reminders has yet to be investigated. Here, we examined the neural correlates that enable social-support-reminders to resist becoming associated with fear during a retardation-of-acquisition test. We found that social-support-figure-images (*vs* stranger-images) were less readily associated with fear, replicating prior work, and that this effect was associated with decreased amygdala activity and increased ventromedial prefrontal cortex (VMPFC) activity for social-support-figure-images (*vs* stranger-images), suggesting that social-support-engagement of the VMPFC and consequent inhibition of the amygdala may contribute to unique their inhibitory effects. Connectivity analyses supported this interpretation, showing greater connectivity between the VMPFC and left amygdala for social-support-figure-images (*vs* stranger-images).

Close social ties play a central regulatory role in our psychological and physiological responses to threat and danger. Knowing that a close other or loved one is nearby can make us feel safe (Bowlby, 1969), mitigate our appraisals of threat (Coan *et al.*, 2006; Eisenberger *et al.*, 2011; Norman *et al.*, 2015) and even diminish our behavioral and physiological responses to danger (Epley, 1974; Thorsteinsson and James, 1999; Hennessy *et al.*, 2009). Recent research has shown that reminders of social-support-figures (i.e. images of close others) spontaneously inhibit the fear-response and belong in a new category of fear inhibitors called ‘prepared fear suppressors’ (Hornstein *et al.*, 2022a) (previously termed “prepared safety stimuli;’ Hornstein *et al.*, 2016; Hornstein and Eisenberger, 2018), uniquely attenuating psychological, neural and physiological responses to perceived threats. Here, we examine the neural correlates that enable social-support-reminders to spontaneously perform one of the central functions of fear inhibitors: resisting becoming associated with fear.

## Unique effects of social support during fear-learning

Social-support-reminders have been shown to spontaneously, such that they require no threat-specific, additional inhibitory-training when introduced to a threatening situation, meet the criteria for the most powerful learned-safety-signals, ‘conditioned inhibitors’ or cues that, through training, come to be associated with the absence of an aversive-event (Rescorla, 1969). Specifically, social-support-reminders spontaneously resist association with fear (to be examined here—retardation-of-acquisition test: Hornstein *et al.*, 2016) and suppress the conditional-fear-response elicited by separate learned-fear cues (summation test: Hornstein *et al.*, 2016; Hornstein *et al.*, 2018), effects that do not occur for images of familiar others (that are not support figures) or positive cues (Hornstein *et al.*, 2016).

Yet, while *conditioned* inhibitors of the fear-response require specific training to pass these tests (Rescorla, 1969; Dickinson and Pearce, 1977; Nasser and McNally, 2012), social-support-figure-reminders meet these criteria without explicit training. Indeed, although there is certainly some amount of learning that takes place during the course of a relationship with a social-support figure during which that individual may provide care, resources and even protection, this previous experience does not mirror the specific safety training required for other fear inhibitors, during which they are learned to inhibit a particular aversive-outcome in a particular context (Rescorla, 1969; Rescorla and Wagner, 1972). For example, a safety signal for shock only becomes so after a participant has been exposed to information that when that stimulus is present in a certain setting, shock is absent in that setting. Yet, it is unlikely that a social support figure has provided protection against electric shock in the laboratory context, and thus the ability of an image of this individual to inhibit fear of shock suggests that this specific training is not required. Certainly, this level of transfer of inhibitory properties across different aversive outcomes and settings is unique. Thus, the ability of social-support-reminders to inhibit fear for novel aversive-events in unfamiliar contexts (Hornstein and Eisenberger, 2018; Hornstein *et al.*, 2022a) distinguishes them from other known fear inhibitors and is unique and noteworthy.

Importantly, social-support-figure-reminders have been shown to have additional unique effects during fear-learning. Specifically, in contrast to previously known fear inhibitors, social-support-figure-reminders can continue to inhibit the fear-response even following their removal, reducing the acquisition of fear (Hornstein and Eisenberger, 2017; Toumbelekis *et al.*, 2018) and enhancing its extinction (Hornstein *et al.*, 2016, 2018; Toumbelekis *et al.*, 2021). While other fear inhibitors reduce fear-responding while present, they have harmful effects in the long-term: augmenting fear-acquisition (Rescorla, 1971; Dickinson, 1976; Leung *et al.*, 2016) and preventing fear-extinction (Rescorla, 1969; Lovibond *et al.*, 2000; Leung *et al.*, 2016). Thus, social-support-reminders not only carry out the inhibitory functions of fear inhibitors without requiring specific training, but also have additional, contrasting, beneficial effects. It is notable that these effects do not appear to occur following instructed fear acquisition (where no aversive outcome, such as shock, is ever experienced: Morato *et al.*, 2021, 2023; Bublatzky *et al.*, 2022), suggesting that the inhibitory effects of social support may not rely on altering expectation that an aversive event will occur, as other fear inhibitors do (Rescorla and Wagner, 1972), but instead rely on altering how aversive the event is perceived to be, as has been argued recently (Hornstein *et al.*, 2022a).

Altogether, the unique characteristics of social-support-reminders—inhibiting fear while present without any specific inhibitory training and bringing about lasting inhibition of fear even following their removal—suggest these reminders fall into a previously unknown category of fear inhibitors, recently labeled prepared fear suppressors (Hornstein *et al.*, 2016, 2022a).^1^ It is possible that the prepared fear suppressor category comprises cues that have historically enhanced mammalian survival such as physical warmth or social support figures. In the case of social support figures, these specific individuals (unique to each person) may be learned, through repeated experiences of care and protection, to meet the requirements for a social support ‘placeholder’ that confers these inhibitory properties (see: Hornstein and Eisenberger, 2018)—enabling these cues to signal security, protection and resources and to engage underlying psychological and neural systems involved in safety-processing and fear-inhibition (Hornstein *et al.*, 2022a).

## Neural mechanisms underlying the effects of social support during fear-learning

Although there is now accumulating evidence that social-support-figure-reminders have unique inhibitory effects during fear-learning processes, little is known about the underlying neural mechanisms that enable these effects. However, research points to two neural regions critical for processes that inhibit fear, including safety-learning (learning that a formerly neutral stimulus comes to predict the absence of an aversive-event) and fear-extinction (learning that a cue does not always lead to an aversive-outcome, which reduces fear-responding to that cue) that may also play a role in the fear inhibiting effects of social-support-figures. Specifically, these two key regions are the amygdala, which is critical for fear-learning, and the ventromedial prefrontal cortex (VMPFC), which inhibits the amygdala during safety-learning and fear-extinction (Delgado *et al.*, 2006; Pape and Pare, 2010).

Animal and human research has established the amygdala as critical for the acquisition and expression of conditional-fears (Davis, 1992; LaBar and LeDoux, 1996). Specifically, stimulation of the amygdala in animals can lead to autonomic and behavioral changes associated with fear (Tellioğlu *et al.*, 1997), whereas amygdala lesions can block these changes (Kapp *et al.*, 1979; McCabe *et al.*, 1992). Moreover, in humans, cues learned to predict an aversive-outcome (conditional-fear-stimuli) activate the amygdala (Büchel *et al.*, 1998; LaBar *et al.*, 1998; Phelps *et al.*, 2004), and patients with amygdala damage fail to show conditional-fear-responses (measured through increases in skin conductance responses (SCRs), an index of sympathetic-nervous-system activity) (Bechara *et al.*, 1995; LaBar *et al.*, 1995). Thus, activity in the amygdala is central to human fear-learning.

While the amygdala is critical for the acquisition and expression of learned-fear, the VMPFC plays an opposite role, detecting safety and inhibiting fear-responses. Specifically, the VMPFC is more active in response to cues that signal safety compared with cues that signal threat (e.g. absence *vs* occurrence of shock, distant *vs* near tarantula) (Phelps *et al.*, 2004; Schiller *et al.*, 2008; Mobbs *et al.*, 2010). The VMPFC is also more active during fear extinction (*vs* conditioning) (Gottfried and Dolan, 2004, Molchan *et al.*, 1994), during extinction learning (Milad *et al.*, 2007) and during extinction recall (Kalisch *et al.*, 2006). Importantly, the VMPFC has inhibitory control over the amygdala, leading to reductions in fear-responses to conditional-fear-cues when safety signals are present or during fear-extinction procedures (Delgado *et al.*, 2006). Indeed, research in rats demonstrates that stimulating the infralimbic (IL) cortex, homologous to the human VMPFC, reduces fear responding (Milad and Quirk, 2002), and that temporarily inactivating the IL cortex blocks the expression of conditional-safety (Kreutzmann *et al.*, 2020) and impairs retrieval of fear-extinction learning (Kim *et al.*, 2016). Similarly, human research has shown that greater VMPFC activity (Phelps *et al.*, 2004; Milad *et al.*, 2007) and greater VMPFC-amygdala connectivity (Javanbakht *et al.*, 2021) lead to more complete fear-extinction. In sum, the VMPFC appears to be critical for the inhibition of fear-responding and may do so by inhibiting amygdala activity.

Importantly, and most relevant to the current study, in addition to being critical for safety signaling and fear-extinction, animal work has demonstrated that the IL cortex is also critical for retardation-of-acquisition effects—the ability of a cue to resist becoming associated with fear (the process examined here). During retardation-of-acquisition, the acquisition of a new association between fear and a cue is delayed. This is caused by competing associations for that cue—in particular, the cue is first imbued with an inhibitory association (i.e. that an aversive-event (e.g. shock) will not occur) during prior inhibitory learning, and then is trained to be associated with an excitatory association (i.e. that an aversive-event (e.g. shock) will occur) during fear-acquisition (Rescorla, 1969). In animal work, rats with IL lesions no longer showed retardation-of-acquisition effects, suggesting that the IL is involved in maintaining inhibitory control in the face of competing excitatory associations associated with the same stimulus (Rhodes and Killcross, 2007). Thus, VMPFC, as the human homologue to IL cortex, may be particularly important for the retardation-of-acquisition test being examined in this study, namely retarding the association of fear with images of social-support-figures.

There is also reason to believe that the VMPFC may play a role during the retardation-of-acquisition that occurs specifically while viewing social-support-reminders. While the neural mechanisms underlying the fear-inhibiting properties of social support during fear-learning have yet to be examined, the more well-investigated process of social buffering can offer important insights. During social buffering, stress and threat responses are reduced by the presence of a companion or close other (Cohen and Wills, 1985; Kikusui *et al.*, 2006; Hennessy *et al.*, 2009). Animal work examining this process has shown that familiar others increase activity in regions of the medial prefrontal cortex (MPFC) (da Costa *et al.*, 2004) and reduce amygdala activity (da Costa *et al.*, 2004; Kiyokawa *et al.*, 2009, 2012). Although few studies have examined these processes in humans (c.f., Coan *et al.*, 2006), a neural investigation showed that viewing images of close others during painful events led to increased VMPFC activity, which was associated with reduced self-reported pain as well as reduced pain-related neural activity (Eisenberger *et al.*, 2011). Together, these clues from the social buffering literature suggest that the inhibitory effects of social-support-reminders during fear-learning may rely on this pathway.

## Current investigation

Because no prior work has examined the specific neural mechanisms that allow social-support-figure-reminders to confer their fear inhibiting effects, we examined the neural mechanisms underlying social-support-driven retardation-of-acquisition. During a retardation-of-acquisition test procedure, images of social-support-figures as well as control images of smiling-strangers (sex-, age-, and gender-matched to the social-support figure) were repeatedly paired with shock while neural and SCR data were collected. Replicating prior work (Hornstein *et al.*, 2016), we hypothesized that images of a social-support-figure paired with shock (*vs* not) would elicit significantly smaller conditional-fear-responses than images of a stranger (control) paired with shock (*vs* not), suggesting that social-support-figure-reminders are less readily associated with the threat of shock. With regard to neural activity, we hypothesized that: (1) images of social-support-figures paired with shock (*vs* not) would elicit significantly less amygdala activity than stranger-images paired with shock (*vs* not), indicating smaller fear-responses, (2) there would be greater VMPFC activity in response to social-support-figure-images *vs* stranger-images, indicating greater detection of safety and (3) consistent with findings that the VMPFC has inhibitory connections with the amygdala, there would be greater functional connectivity between the VMPFC and amygdala when social-support-figure-images (*vs* stranger-images) were paired with shock (*vs* not), indicating greater fear-inhibition.

## Methods

### Participants

All participants (*N* = 80) were recruited from the University of California Los Angeles (UCLA) campus and surrounding area via flyers. The UCLA Institutional Review Board approved all study procedures. Participants provided written informed consent. Of these participants, 25 ultimately declined to attend the scanner session after completing the initial screening. Of the remaining participants (*N* = 55), a final sample of 31 was used for behavioral analyses and 42 were used for imaging analyses meeting the target sample size of *N* = 30 based on a-priori power analyses (see Supplemental Information (SI) for details).

### Procedure overview

All participants underwent the same procedures, which took place in the UCLA Psychology Department and then at the UCLA Brain Mapping Center.

### Screening procedures

#### Telephone screening

Following a telephone screening, participants were excluded from participating if they were pregnant, had any history of mental illness, were currently taking any mental health-related medications, had any metal in their bodies, were left-handed or were claustrophobic.

#### SCR Screening

Eligible participants arrived at the lab in the Psychology Department for a 30 minute screening procedure to determine if their SCR could be detected by the experimental equipment (please see SI for details), and only participants whose SCR could be detected were enrolled and able to continue on in the experiment (*N* = 80).

If eligible, participants were asked to identify ‘the two individuals who give you the most social support on a daily basis,’ where social support was defined as: ‘someone you can call if you are having a bad day or when you have good news to share.’ Participants rated how much average social support they received daily from each individual on a scale of 1–10 (where 1 = ‘no social support’ and 10 = ‘a lot of social support’: *M = *9.04*, SD = *0.84). Participants were required to send a digital photograph of each of their social-support-figures to the experimenter prior to their experimental session (participants selected and provided these photographs from within their own digital photographs and were instructed to choose images in which their social-support-figures were facing the camera with all features visible). If they were unable to identify a close individual rated as providing support at a 7 or higher on the scale or were unwilling to send a photograph, they were excluded from further participation.

### Functional magnetic resonance imaging task design and image acquisition

#### Shock calibration procedure

Upon arriving at the UCLA Brain Mapping Center, participants underwent a shock-calibration procedure (outside the scanner) to determine the individual level of shock to be used during the experiment (please see SI for details).

#### Experimental procedure

Once in the scanner (please see SI for details), participants underwent a fear conditioning procedure with three stages: habituation, acquisition and extinction. During each stage, two sets of stimuli were presented. One set included two images of social-support-figures (selected, rated and provided by participants: social-support-condition) and the other set included two images of strangers (stranger-condition). Images in the stranger condition were found by conducting an online search for free images of individuals who were matched to the individual in the social support figure image in terms of gender, relative age, ethnicity, hair-color, and other accessories or noticeable features (i.e. glasses, hearing-aids, piercings, etc.). During all stages, images were presented for 6 seconds followed by a 14 second inter-stimulus-interval (ISI) before the next image presentation. Fixed ISIs were used here to mirror previous work from this group and others (Hornstein *et al.*, 2016; Hornstein and Eisenberger, 2018; Toumbelekis *et al.*, 2018) and to ensure exact comparisons across stimulus conditions by trial, but future work should investigate similar procedures with variable ISIs. Image presentations were made in one of two pseudorandom orders (each designed to ensure that shock-trials were never applied more than twice in a row) that were counterbalanced across participants. SCR was collected during all stages of the experiment.

**Fig. 1.**
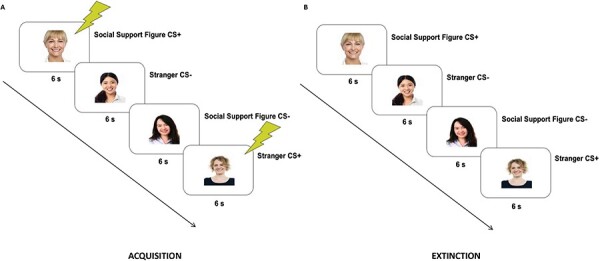
Example of experimental trials. **A.** During the acquisition stage, one CS of each type (social-support-figure-image, stranger-image) was continuously presented with a co-terminating 500 ms electric shock (CS + s) and one CS of each type was never paired with shock (CS-). **B.** During the extinction stage, all CSs were presented on their own, in the absence of shock. Example images in figure courtesy of stockimages and posterize at FreeDigitalPhotos.net.

After undergoing a Habituation-stage (see SI for details) to ensure that no pre-existing characteristics of any stimulus would account for later effects, participants underwent an Acquisition-stage. During the Acquisition-stage, participants saw 10 presentations of each image. One image from each condition was consistently paired with a co-terminating 500 ms mild electric shock (CS+ images: 100% reinforcement schedule) and the other image from each condition was never paired with shock (CS- images). This differential learning procedure allowed us to compare SCR for CS + s to CS-s within each condition to assess whether a conditional-fear-response was acquired (indicated by significantly higher SCR for a CS+ *vs* CS- from the stimulus-type) (Figure 1A).

Following the Acquisition-stage, participants had a short break during which they watched a brief video clip about airplanes. After this came the Extinction-stage, during which participants viewed six non-reinforced presentations of each image (Figure 1B). The first two trials of this stage served as a second test of conditional-fear-acquisition, and the final trials were designed to extinguish any conditional-fear acquired.

#### Post-experimental procedure follow-up

After participants exited the functional magnetic resonance imaging (fMRI) scanner, they were asked which images were paired *vs* not paired with shock, allowing us to assess whether they were aware of the shock contingencies and their level of attention during the procedures.

## Analytic overview

### Behavioral analyses

#### SCR Data Pre-processing

SCR data were preprocessed according to current recommendations (Figner and Murphy, 2011; Lonsdorf *et al.*, 2017: see SI for details). Before data were analyzed, we determined whether each participant met two requirements. First, we determined whether participants reported awareness of the shock-stimulus pairing during the post-experimental session follow-up and excluded any participant unaware of these contingencies (*N* = 3). This was done to exclude cases in which participants were unable to discriminate between the stimuli or were not able to pay adequate attention, leading to unawareness of contingencies (see: Dawson and Schell, 1985). Next, we evaluated whether participants were low responders (*N* = 6; see SI for details). This was done in order to exclude cases in which it was unclear if low numbers of responses were due to low levels of learned-fear or due to lack of attention or distraction in the fMRI environment. All exclusion criteria were determined based on previous work and current recommendations for SCR data collection and processing (Olsson *et al.*, 2005; Schiller *et al.*, 2010; Figner and Murphy, 2011; Hornstein *et al.*, 2016, 2017, 2018; Lonsdorf *et al.*, 2017).

#### SCR data analyses

In order to assess fear-acquisition, we ran a 2 × 2 × 2 within-subjects ANOVA to assess SCR across condition (social support, stranger), reinforcement (reinforced, nonreinforced) and time (first 5 trials, last 5 trials). The 10-trial acquisition procedure was divided into early and late phases to examine whether there were differences in SCR during early trials, which were used to assess the periods of greatest associative change, and late trials, which were used to assess the product of that learning. Because we had no specific hypotheses regarding interactions with time, all analyses with time were FDR-corrected.

Next, using the acquisition means, we ran post-hoc tests to assess whether fear-responses were acquired in each condition, indicated by significantly higher SCR for the CS+ *vs* the CS- within each condition (social support, stranger). Because we had specific predictions that the difference between the CS+ *vs* CS- would be greater for stranger *vs* social support images, we did not correct for multiple comparisons within these post-hoc tests.

We conducted the same set of analyses (but without time as a variable) using the first two trials from the extinction-stage to assess whether differences in learning persisted beyond the acquisition-stage. Because this study used a 100% reinforcement schedule, conditional fear responses extinguish quickly and thus, we only examined SCR during the first two trials of each condition. SCR during the first two trials of extinction served as a second test of conditional-fear-acquisition, measuring fear-responding to each CS+ after the acquisition procedure was complete and time had elapsed since the most recent shock was applied (as used in previous work: Hornstein *et al.*, 2016, 2017, 2018).

### Neuroimaging analyses

#### fMRI data acquisition and analysis

Details regarding fMRI data acquisition and analysis can be found in the SI.

#### ROI analyses

Details of ROI creation can be found in the SI. For each ROI, we ran a 2 × 2 × 2 within-subjects ANOVA to assess neural activity across condition (social support, stranger), reinforcement (shock, no shock) and time (first 5 trials, last 5 trials). For the amygdala ROI, we hypothesized a condition × reinforcement interaction, such that there would be greater differences in amygdala activity to stranger CS+ *vs* CS- images compared to social support CS+ *vs* CS- images. Because we had specific hypotheses regarding the pattern of this interaction, we did not employ corrections for multiple comparisons within the post-hoc analyses of the interaction. For any effects for the bilateral amygdala ROI, we further investigated whether these effects held for the left and right amygdala separately. For the VMPFC ROI, we hypothesized a main effect of condition such that there would be more VMPFC activity during all social support trials compared to all stranger trials. All analyses were thresholded at *P* < 0.05, two-tailed. Because we had no specific predictions regarding interactions with time or effects for left and right amygdala separately, these analyses were FDR-corrected.

#### Connectivity analyses

ROI-based functional connectivity analyses were conducted using ROI-to-ROI analyses to determine functional connectivity (i.e. temporal correlations) between the VMPFC and amygdala (see SI for details). Here too, we ran a 2x2x2 within-subjects ANOVA to assess connectivity across condition (social support, stranger), reinforcement (shock, no shock), and time (first 5 trials, last 5 trials). We hypothesized an interaction between condition and reinforcement. Because we had specific hypotheses regarding the pattern of this interaction, we did not employ corrections within the post-hoc analyses of the interaction. We further explored connectivity with left and right amygdala separately (FDR-corrected). Analyses were thresholded as described above.

## Results

### Do social-support-figure-images retard fear-acquisition?

**Fig. 2.**
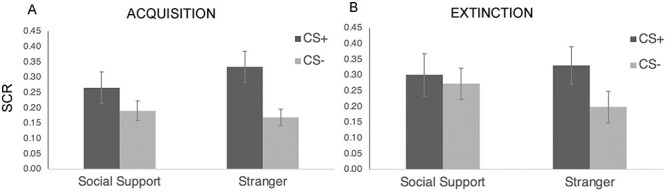
SCR results from the: **(A)** entire acquisition phase and **(B)** first two trials of extinction broken down by condition (social support, stranger) and reinforcement (CS+, CS-). All error bars indicate standard error.

We first examined whether social-support-figure-reminders were less readily associated with fear by examining the hypothesized condition × reinforcement interaction. Replicating prior work (Hornstein *et al.*, 2016), we found a significant condition × reinforcement interaction (*F*(1,30) = 10.20, *P *= 0.003, η_p_^2^ = 0.25), such that SCR to social-support-figure-images paired with shock (*vs* not) were significantly lower than SCR to stranger-images paired with shock (*vs* not) (Figure 2A; see SI for trial-by-trial data). Thus, while subjects acquired conditional-fear-responses to both the social-support-figure-images (F(1,30) = 5.16, *P* = 0.03, η_p_^2^ = 0.15) and the stranger-images (F(1,30) = 22.10, *P* < 0.001, η_p_^2^ = 0.42), conditional-fear-responding to the social-support-figure-images was significantly weaker. Indeed, SCR to the social-support-figure-images (CS+ *vs* CS-) were about two times lower than SCR in response to the stranger-images.^2^ In addition, there was a main effect of reinforcement, such that trials paired with shock showed larger SCRs than trials not paired with shock (F(1,30) = 14.79, *P* < 0.001, η_p_^2^ = 0.33) and a main effect of time, such that early trials showed larger SCRs than late trials (F(1,30) = 93.28, *P* < 0.001, FDR-corrected: *P* = 0.004, η_p_^2^ = 0.76). There were no other main effects or interactions with time (*P* > 0.36).

Importantly, these patterns of learning persisted beyond the acquisition period. Specifically, when examining the first two trials of the extinction-stage (another measure of the strength of acquisition), we found that SCR to social-support-figure-images paired with shock (*vs* not) were still significantly lower than SCR to stranger-images paired with shock (*vs* not) (*F*(1,30) = 6.27, *P *= 0.018, η_p_^2^ = 0.17) (Figure 2B). Specifically, we found that while subjects still showed a significant conditional-fear-response to stranger-images (*F*(1,30) = 13.41, *P* < 0.001, η_p_^2^ = 0.31), there was no longer a conditional-fear-response to social-support-figure-images (F(1,30) = .44, *P *= 0.51, η_p_^2^ = 0.01; replicating Hornstein *et al.*, 2016), suggesting that the fear-association for the social-support-figure-images was not only weaker during acquisition but also less able to endure beyond the end of the acquisition procedure. In addition, there was a main effect of reinforcement, such that trials paired with shock showed larger SCRs than trials not paired with shock (F(1,30) = 5.86, *P* < 0.05, η_p_^2^ = 0.16), but no main effect of condition (p >0.56).

### What are the neural regions underlying social-support-associated retardation-of-acquisition?

#### Amygdala activity

**Fig. 3.**
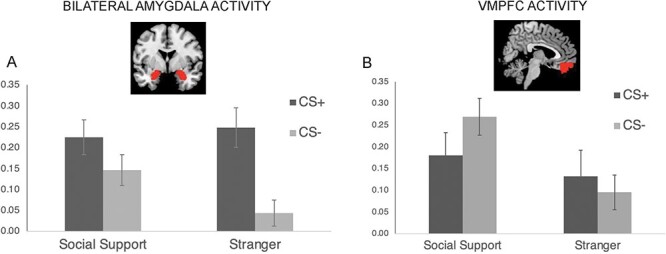
Neural results from the acquisition phase. All error bars indicate standard error. (**A)** Parameter estimates for the bilateral amygdala ROI broken down by condition (social support, stranger) and reinforcement (CS+, CS-). (**B)** Parameter estimates for the VMPFC ROI broken down by condition and reinforcement.

Given the central role of the amygdala in fear-learning, we focused on neural activity in a bilateral amygdala ROI during the condition × reinforcement interaction. ROI analyses confirmed a condition × reinforcement interaction, (*F*(1,41) = 4.50, *P *= 0.04, η_p_^2^ = 0.10) (Figure 3A). Post-hoc analyses revealed that, while there was no significant difference in amygdala activity to social-support-figures in response to the CS+ *vs* CS- (*F*(1,41) = 2.23, *P *= 0.14, η_p_^2^ = 0.05), there was significant activation in the amygdala in response to stranger-images during CS+ *vs* CS- (*F*(1,41) = 14.90, *P *< 0.001, η_p_^2^ = 0.27), mirroring the patterns of SCR responding. In addition, there was a significant main effect of reinforcement (F(1,41) = 10.50, *P* = 0.002, η_p_^2^ = 0.20), such that trials paired with shock showed greater amygdala activity than trials not paired with shock, and a marginally significant main effect of condition (F(1,41) = 3.89, *P* = 0.06, η_p_^2^ = 0.09), such that trials paired with social support figures showed greater amygdala activity than trials paired with strangers. There were no other significant effects (*P* > 0.29).

We also examined the left and right amygdala separately. Because there were no significant interactions with time for the previous analysis, time was taken out of the model. There was a significant condition by reinforcement interaction for the left amygdala (*F*(1,40) = 4.74, *P *= 0.035, FDR-corrected: *P* = 0.039, η_p_^2^ = 0.11), but this effect did not survive FDR-correction for the right amygdala (*F*(1,41) = 4.34, *P *= 0.043, FDR-corrected: *P* = 0.06, η_p_^2^ = 0.10). Post-hoc analyses of the left amygdala revealed that, as expected, while there was no significant difference in left amygdala activity to social-support-figures in response to the CS+ *vs* CS- (*F*(1,40) = 3.38, *P *= 0.07, η_p_^2^ = 0.08), there was significant activation in the left amygdala in response to stranger-images during CS+ *vs* CS- (*F*(1,40) = 21.96, *P *< 0.001, η_p_^2^ = 0.35). In addition, there was a significant main effect of reinforcement for the left and right amygdala (left: *F*(1,40) = 15.18, *P *< 0.001, FDR-corrected: p = 0.003; η_p_^2^ = 0.28; right: *F*(1,41) = 8.14, *P *= 0.007, FDR-corrected: p = 0.021; η_p_^2^ = 0.17), such that amygdala activity was greater for trials paired with shock *vs* not. There was also a significant main effect of condition for the left amygdala (*F*(1,40) = 4.53, *P *= 0.039, FDR-corrected: *P* = 0.039; η_p_^2^ = 0.10), such that amygdala activity was greater during social-support (*vs* stranger) trials, but not for the right amygdala (*F*(1,41) = 3.23,*P *= 0.08, FDR-corrected: *P* = 0.08; η_p_^2^ = 0.07).

#### VMPFC Activity

We next examined the role of the VMPFC. Because the VMPFC is known to respond to social-support-figure-images broadly (Eisenberger *et al.*, 2011), we hypothesized a main effect of condition (social support *vs* stranger) on VMPFC activity because social-support-trials should activate the VMPFC, regardless of shock, therefore leading to similar activity across shock and non-shock paired trials. As expected, we found a main effect of condition (F(1,41) = 15.68, *P* < 0.001, η_p_^2^ = 0.28), such that there was significantly more VMPFC activity in response to social-support-figure *vs* stranger-images, suggesting that social-support-reminders increase VMPFC activity (Figure 3B). There was no main effect of reinforcement (p = 0.57) or time (*P* = 0.08, FDR-corrected: *P* = 0.13) and no condition x reinforcement interaction (*P* = 0.14). In addition, the exploratory condition x reinforcement x time interaction (F(1,41) = 4.50, *P* = 0.04, η_p_^2^ = 0.10) did not survive FDR-correction (*P* = 0.13).

#### Connectivity between VMPFC and amygdala

Given the important role of the VMPFC in inhibiting amygdala activity during fear-extinction processes (Phelps *et al.*, 2004), we next examined VMPFC-amygdala connectivity to explore whether the relationship between these two regions might be critical for retarding fear-acquisition through processes similar to those observed during fear-extinction.

When examining the condition × reinforcement interaction for VMPFC-bilateral amygdala connectivity, there was not significantly greater connectivity during social support (CS+ *vs* CS-) *vs* stranger (CS+ *vs* CS-) images (*F*(1,41) = 2.66, *P *= 0.11, η_p_^2^ = 0.001). No other effects were significant (*P* >0.36).

**Fig. 4.**
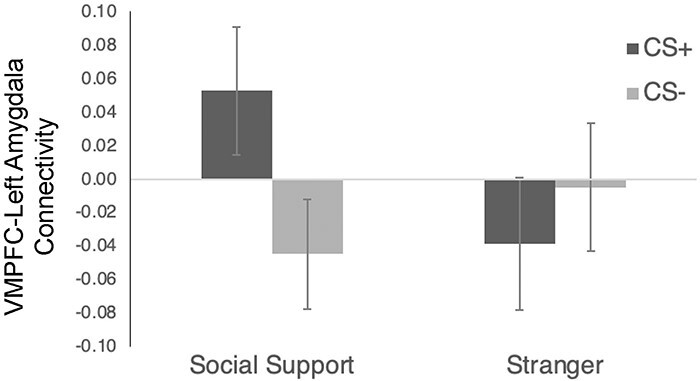
VMPFC-left amygdala connectivity results from the acquisition phase broken down by condition (social support, stranger) and reinforcement (CS+, CS-). All error bars indicate standard error.

However, because analyses of the bilateral amygdala revealed that the social support-related effects were significant for the left, but not the right, amygdala, we further explored connectivity with left and right amygdala separately (time was dropped as a variable because it was not significant in the previous analyses). Thus, there was a significant condition x reinforcement interaction for VMPFC-left amygdala activity (F(1,41) = 9.86, *P* = 0.003, FDR-corrected: *P* = 0.009; η_p_^2^ = 0.19), such that VMPFC-left amygdala connectivity was greater during CS+ *vs* CS- trials for social support images (F(1,41) = 7.51, *P* = 0.009, η_p_^2^ = 0.16), but there was no difference for stranger images (F(1,41) = 1.27, *P* = 0.27, η_p_^2^ = 0.03) (Figure 4). There were no other effects for VMPFC-left amygdala connectivity (*P* > 0.21). There were also no effects for VMPFC-right amygdala connectivity (*P* > 0.27). Thus, in sum, there was significant connectivity between VMPFC and left amygdala during the social-support-trials (CS+ *vs* CS-) but not during the stranger-trials. This is consistent with the amygdala findings in which the buffering effects of social support on fear learning were significant for the left, but not the right, amygdala. Given that most VMPFC projections to the amygdala are excitatory (Rosenkranz and Grace, 2001), it has been suggested that VMPFC inhibition of the amygdala occurs by activating inhibitory neurons within the amygdala (Quirk and Gehlert, 2003; Rosenkranz *et al.*, 2003). Therefore, positive connectivity between these regions during this learning period may have been involved in reducing the strength of conditional-fears in the social-support-condition.

## Discussion

The unique ability of social-support-reminders to both inhibit fear while present as well as to reduce fear in the long-term (Hornstein *et al.*, 2022a) renders investigations into the neural underpinnings of these effects as crucial. In particular, before exploring the clinical benefits of social-support-figure-reminders for reducing fear, understanding the mechanisms through which these effects are achieved is paramount.

The results of the current work replicate previous findings showing that social-support-figure-reminders retard fear-acquisition (*vs* stranger controls: Hornstein *et al.*, 2016). They further reveal differential neural activity during the inhibition of fear by social-support-reminders. In particular, VMPFC activity was increased for images of social-support-figures, regardless of their role in the fear-learning context—likely due to their inherent characteristics that signal support and care—and amygdala activity, particularly left amygdala activity, was reduced for social-support-figure-images (*vs* stranger-images) paired with an aversive-outcome, perhaps leading to the weaker fear-associations observed for these cues. Additionally, there was significantly more VMPFC-left amygdala connectivity in response to social-support-images paired with shock (*vs* not) compared to stranger-images paired with shock (*vs* not), indicating that communication between these regions may be critical for the reduced conditional-fear-response observed to social-support-images paired with shock (*vs* not). Thus, the process by which social-support-figure-reminders retard fear may rely on engagement of VMPFC that consequently inhibits left amygdala activity.

Interestingly, the fact that the observed effects for the bilateral amygdala were significant for the left, but not the right, amygdala is consistent with some prior work showing a relationship between social support and left amygdala function. For example, prior work has shown that greater perceived social support was associated with greater left, but not right, amygdala volume (Sato *et al.*, 2016). Greater perceived social support has also been shown to be associated with increased connectivity between the left amygdala and right orbitofrontal cortex during a resting state scan (Sato *et al.*, 2020). Finally, other work has shown a buffering effect of social support on anxiety in the left amygdala; while greater left amygdala activity is related to greater levels of anxiety in those with lower levels of social support, for those with high levels of social support, this relationship is non-significant (Hyde *et al.*, 2011). While only speculative at this point and these results should be interpreted with caution (as these lateralized effects were not hypothesized in advance), it is possible that the left amygdala plays a stronger role in the buffering effects of social support. Further research is needed to better understand these findings.

Although this work only investigates the neural mechanisms underlying the ability of social-support-reminders to bring about retardation-of-acquisition, there is reason to believe similar neural correlates may be responsible for the other unique inhibitory effects of social support. Indeed, retardation-of-acquisition occurs due to competing inhibitory and excitatory associations for the same cue, ultimately resulting in reduced conditional-fear-acquisition. This process is the reverse of the more widely known inhibition process that occurs during fear-extinction, in which a stimulus is first imbued with an excitatory association and then with an inhibitory association during the extinction procedures (Rescorla and Wagner, 1972), and is separate from the other test of a conditioned inhibitor, summation, during which two distinct stimuli, one with an excitatory association and one with an inhibitory association, are paired (Rescorla, 1969). Yet all three of these processes involve inhibition of the fear-response and likely rest on similar underlying processes. Therefore, while further work should explore the neural mechanisms underlying each distinct processes, the unique ability of social-support-figure-reminders to retard acquisition during retardation-of-acquisition, suppress fear-responding during summation and augment fear-extinction outcomes may rest on their ability to engage the VMPFC and consequently inhibit activity in the amygdala.

This potential pathway is notable, as the VMPFC is not only central to safety-learning and fear-extinction processes, but it has also been shown to have altered responding to threats in those with anxiety and fear related disorders. Specifically, those with anxiety disorders exhibit decreased VMPFC activity in response to safety or extinction learning, decreased VMPFC volume and increased VMPFC activity in response to inappropriate (threatening) cues (Hartley and Phelps, 2012; Cha *et al.*, 2014; Grego and Liberzon, 2016). Additionally, altered VMPFC activity is thought to be one factor that impedes the success of exposure therapies, the most successful treatment for anxiety disorders to date. Indeed, these treatments engage fear-extinction processes to reduce dysfunctional fear symptoms (Hermans *et al.*, 2006; Craske *et al.*, 2018)—yet, despite their relative effectiveness, relapse and return of fear remain common, perhaps due to decreased VMPFC activation in those with dysfunctional fears (Grego and Liberzon, 2016; Maren and Holmes, 2016; Craske *et al.*, 2018). Thus, if social-support-reminders are able to engage the VMPFC due to their supportive characteristics (regardless of the presence or absence of threat) and consequently impact fear-learning processes, they may represent a useful method to increase VMPFC activity, and corresponding fear-inhibition, in anxious groups.

Still, more work is required to fully understand the neural underpinnings of the inhibitory effects of social-support-figure-reminders. Notably, the current work examines neural activity that occurs when social-support-figure-reminders retard fear-acquisition, but future work must examine whether similar pathways underlie the ability of social-support-reminders to suppress fear (summation) and enhance long-term fear-extinction outcomes (Hornstein *et al.*, 2016, 2018). Additionally, given the known differences in VMPFC activity in individuals with anxiety disorders, future work must examine whether similar patterns of neural effects occur for social-support-reminders in those with diagnosed anxiety disorders.

In combination with growing awareness of the distinct inhibitory properties of social-support-figure-reminders, the results of the current work contribute to a greater understanding of the novel prepared fear suppressor category (Hornstein *et al.*, 2022a). This emerging understanding not only expands the fear inhibitor classification, but also has the potential to inform approaches to reduce dysfunctional fears. Notably, social-support-figure-reminders, or perhaps other prepared fear suppressors, may represent a before-untested, and relatively non-invasive, method to boost current exposure therapy treatments, improving relief from the harmful effects that induce pathological fears.

## Supplementary Material

nsae002_Supp

## Data Availability

All data from the work reported in this manuscript can be found on the Harvard Dataverse (https://doi.org/10.7910/DVN/OCMQIT).

## Footnotes

1 Notably, physical warmth has also been shown to be a member of this category, with physically warm objects performing the same combination of unique inhibitory functions as social-support-reminders (Hornstein *et al.*, 2022b).

2 It should be noted that while in Hornstein *et al.* (2016), there was no acquisition of conditional-fears in the social-support-figure-condition, unlike here, those procedures were conducted in a behavioral laboratory environment which was far less stressful than the fMRI environment in which this current work was conducted. The added stress of being in the confines of the fMRI scanner may have contributed to the ease with which fear was acquired.
