# Supplementary material for: Social support and fear-inhibition: an examination of underlying neural mechanisms
Source: Soc Cogn Affect Neurosci. 2024 Jan 12;19(1):nsae002. doi: 10.1093/scan/nsae002 (PMC10868130; doi:10.1093/scan/nsae002)
Supplement: nsae002_Supp [file nsae002_supp.zip › SAS_Supplemental_Information-Final-with_figures.docx]

Social Support and Fear-inhibition:

An examination of underlying neural mechanisms

Hornstein, E.A., Leschak, C.J., Parrish, M.H., Byrne-Haltom, K.E., Fanselow, M.S., Craske, M.G., & Eisenberger, N.I.

**Supplemental Information**

*Methods*

**Participants**

Of the 80 participants enrolled in the study, 55 participants returned to attend the scanner session. Of these, certain participants were excluded from data analysis for the following reasons: 2 participants withdrew from participating, 2 participants were excluded from all analyses due to procedural errors (incorrect conditions applied, shocks not applied), 3 participants were excluded from all analyses because they were unaware of experimental contingencies (see below for more discussion of this issue), 6 participants were excluded from all analyses due to technical malfunctions (e.g., scanner cancellations, corrupted brain response files), 5 participants were excluded from only behavioral analyses due to Skin Conductance Response recording equipment malfunctions, and 6 participants were excluded from only behavioral analyses for being low responders (see below for more discussion of this issue).

*Sample Size Determination*. Power analyses conducted using G*Power software and using data from similar work (examining the effects of social support during two retardation-of-acquisition procedures: Hornstein, Fanselow, & Eisenberger, 2016) indicated that the effects of interest (whether retardation-of-acquisition occurred: Cohen d’s > .69; Cohen’s f’s > .46) could be replicated at greater than 95% power (α=.05, two-tailed) using sample sizes between 19-30 (depending on the analysis). Hence, the current work included 42 participants who attended the scanning session (all data used in neuroimaging analyses) in order to reach the required 31 participants for whom data could be analyzed to examine behavioral fear conditioning outcomes (please see above for why 11 participants were excluded from behavioral data analyses).

**Skin Conductance Response (SCR) Screening**

*Equipment.* During both the SCR screening and the imaging experimental session, SCR was collected using AcqKnowledge 3.9 software along with a BioPac MP100 system with EDA Radiotranslucent Dry Electrodes made for use in the MR environment (up to 7T with any scanning sequence) and Isotonic Recording Electrode Gel (BioPac Systems, Inc., Aero Camino Goleta, CA). All recordings were collected with waveform and acquisition sample rates of 1000 Hz per second.

*Screening Test.* Electrodes were placed on the palmar side of the medial phalanges on the fore and middle fingers of participants’ left hands. Participants were then instructed to take deep, evenly spaced breaths, actions that should activate the sympathetic nervous system and consequently lead to measurable increases in SCR. An experimenter monitored SCR responses to determine if the equipment could detect these increases.

**fMRI Task Design and Behavioral Data Acquisition**

*Equipment.* SCR data was collected and tested as described above. Shocks were applied using BioPac Radiotranslucent Electrodes with Electrolyte Gel (10% chloride salt) made for use in the MR environment (up to 7T with any scanning sequence) connected to a Digitimer Constant Current Stimulator (model DS7AH, Digitimer North America, LLC, Fort Lauderdale, FL) connected to an amplifier built into the BioPac MP100 system. Shocks were signaled using E-prime 2.0 professional software. During the experimental procedure, instructions, images, and fixation crosses were also presented using the E-Prime 2.0 software (Psychology Software Tools, Inc., Sharpsburg, PA, 15215).

*Shock Calibration Procedure*. Once the shock electrodes were applied to participants’ right biceps, 500ms shocks were applied starting at 1 mA (1000 microsiemens (μS), 400 Volts) and increasing in .5 mA increments up to 6 mA. Participants were instructed to alert the experimenter when the applied shock was experienced as “extremely uncomfortable, but not yet painful,” a calibration procedure used in previous studies by this (Hornstein, et al., 2016; Hornstein & Eisenberger, 2017; Hornstein, et al., 2018) and other teams (Olsson, et al. 2005; Phelps, et al., 2004; Schiller, et al., 2010). Once a level of shock that was experienced as uncomfortable but not yet painful was identified by the participant, this level of shock was used throughout the experiment. Average mA was 4.6 mA for all participants who attended the scanner session (except for 2 who withdrew before the calibration procedure and one for whom the scan was canceled: n = 52), 4.73 mA for participants included in behavioral (SCR) analyses (n = 31), and 4.67 mA for participants included in fMRI data analysis.

*Experimental Procedure.* Upon entering the fMRI environment, participants were asked to lay down on the scanner bed and leads connecting individual electrodes placed on the participants’ skin to the SCR and shock equipment were applied. A mirror was attached to the head coil so that participants could view a projector screen located behind the scanner. Once moved into the scanner, participants were instructed to pay attention to the projector screen upon which they would be viewing different visual stimuli.

For the first stage of the experiment, participants underwent a habituation procedure (Habituation stage). During this, participants saw four non-reinforced (no shock) presentations of each image (16 presentations total). This allowed us to assess whether any stimulus had pre-existing characteristics that led it to arouse more SCR than the other stimulus in its condition. No such pre-existing characteristics were found (p’s> .552, see below for full results), therefore later differences in SCR for stimuli within each condition can be attributed to effects of the acquisition procedure.

**fMRI Data Acquisition and Analysis**

*Equipment.* Neuroimaging data were acquired on a Siemens Prisma 3.0 Tesla MRI scanner at the UCLA Brain Mapping Center. Head movements were restrained with foam padding. For each participant, a T1-weighted magnetization prepared rapid gradient echo (MPRAGE) anatomical image (slice thickness = 0.9 mm, 192 slices, TR = 2300 ms, TE = 2.32 ms, flip angle = 8°, matrix = 256×256, FOV = 240 mm) was acquired coplanar with the functional scan. During the acquisition stage, one functional scan was acquired (echo-planar T2*-weighted gradient-echo, 401 volumes, slice thickness = 3 mm, gap = 1 mm, TR = 2000 ms, TE = 24 ms, flip angle = 90°, matrix = 64×64, FOV = 200 mm).

*Data Analysis.* Neuroimaging data were analyzed using Statistical Parametric Mapping (SPM12; Wellcome Department of Cognitive Neurology, Institute of Neurology, London). Images were realigned to correct for head motion, normalized to Montreal Neurological Institute (MNI) space using diffeomorphic anatomical registration through exponentiated lie algorithms (resampled at 3×3×3 mm), and spatially smoothed using a 5 mm Gaussian kernel, full width at half maximum, to increase signal-to-noise ratio. General linear models (GLMs) were constructed for each participant, and linear contrasts of interest were computed. The time series was high-pass filtered (128 Hz) and serial autocorrelation was modeled as an autoregressive AR(1) process.

MRI data were pre-processed with the Statistical Parametric Mapping software (SPM12; Wellcome Department of Cognitive Neurology, London, UK). The pre-processing pipeline incorporated image realignment to correct for head movement, co-registration of the functional to the structural images, and Diffeomorphic Anatomical Registration using Exponentiated Lie algebra (DARTEL)-based spatial normalization to Montreal Neurologic Institute (MNI) space (resampled at 3 mm isotropic), and spatial smoothing using a 5mm Gaussian kernel, full width at half maximum, to increase signal-to-noise ratio. Motion and outlier censoring was conducted with custom lab-based scripts (global signal z-threshold = 2.5; translation or rotation motion threshold = 3 mm).

Trials were split into early and late periods of acquisition in the same way as the SCR trials, such that the first 5 presentations of each stimulus were modeled as early learning and the last 5 presentations of each stimulus were modeled as late learning. All four images (social support figure CS+, social support figure CS-, stranger CS+, and stranger CS-) were modeled in separate regressors. For these images, the first 5 seconds of presentation was included in the regressor, while the last 1 second (which contained shock for CS+ images) was modeled in a separate set of regressors. The ISI between image presentations was not explicitly modeled and contributed to the implicit baseline.

The VMPFC ROI was manually constructed in FSLview in a voxel-by-voxel fashion, informed by meta-analyses and reviews pertaining to MPFC function (Northoff, et al., 2006): the VMPFC ROI was constrained to the following: −10 < x < 10, 12 < y < 70, −34 < z < −12. The bilateral amygdala ROI was generated from the Automated Anatomical Labeling (AAL) Atlas (Tzourio-Mazoyer et al., 2002). Mean parameter estimates were extracted for each ROI and entered into standard statistical software (SPSS 25) for further analysis.

**Analytic Overview**

**Behavioral Analyses**

*SCR Data Pre-processing.* SCR data was preprocessed according to current recommendations (Figner & Murphey, 2011; Lonsdorf, et al., 2017). First a low-pass filter, with the cutoff frequency for the low-pass filter being calculated as our sampling rate divided by 8 (in this case, ending in a final cutoff of 125Hz), and smoothing were applied. Then peak-to-peak amplitude in μS was evaluated for each trial (stimulus presentation) by measuring the largest response that occurred between .5-4.5s after stimulus onset. In order to normalize these measurements, square root transformations (as opposed to participant-wise transformations such as z-scoring) were used, a choice that was made for two reasons: 1) to retain the entire range of variability in responding across participants (for a discussion of this issue, see: Figner & Murphy, 2011; Bradford, et al., 2015; Lonsdorf, et al., 2017) and 2) to most closely mirror the analytic methods used in the previous behavioral study demonstrating the ability of social support figures to retard fear acquisition (Hornstein, et. al., 2016), which did not employ such corrections. Given that range corrections of this type are not consistently applied (Figner & Murphy, 2011; Lonsdorf, et al., 2017), we believed these reasons outweighed the benefits of range correction. If a trial occurred during which there was no peak (no rise in SCR during the .5s-4.5s response window) or the peak-to-peak amplitude measure did not meet the threshold of .02 μS, the trial was scored as a zero-response trial. If movement occurred during the trial, as noted by the experimenter during the experimental session, that trial was excluded from analysis.

*Low Responder Determination.* If participants did not show SCR peaks on more than 15% of all trials (<6 trials) during the acquisition stage, at the beginning of which they were informed shock would be paired with certain images and during which shock was applied on 50% of the trials, they were considered low responders and their data was excluded from analysis (n = 6) (all exclusion criteria were modeled on current recommendations and practice for SCR data collection and processing: Hornstein, et al, 2016; Hornstein & Eisenberger, 2017; Hornstein, et al., 2018; Olsson, et al., 2005; Schiller, et al., 2010; Figner & Murphy, 2011)

**fMRI Analyses**

*Connectivity Analyses.* ROI-based functional connectivity analyses were conducted with the CONN toolbox (nitrc.org/projects/conn) with MATLAB and SPM12 software. Pre-processed functional and structural MRI data with condition onsets and durations were imported from the SPM12 univariate GLM models. Confounding variables that distort functional connectivity values were removed through the CONN CompCor algorithm for physiological noise as well as temporal filtering (*f* > .008Hz). Realignment parameters and scrubbing dummy coded variables (representing head movement and global signal outliers) produced during pre-processing were also entered in the toolbox as nuisance covariates to be excluded from analyses. For the BOLD data collected during the fear conditioning task, condition onsets and duration were specified in the toolbox, so that BOLD time series could be appropriately segmented into task-specific blocks. For primary statistical analyses, we conducted ROI-to-ROI analyses to determine functional connectivity (i.e., temporal correlations) between the VMPFC and bilateral amygdala. Effects were then followed up with tests of VMPFC connectivity with the left and right amygdala separately. Within the ROIs, the BOLD signal time series was averaged across all voxels. Connectivity values underwent Fisher’s r-to-Z transformation to ensure assumptions of normality.

*Results*

**Habituation Results**

Results from the habituation stage showed no significant differences across stimuli that were to become CS+s and stimuli that were going to become CS-s within each condition—indicating that no pre-existing characteristics separated future CS+s and CS-s and accounted for later differences in SCR. Specifically, results of a 2 (condition: social support vs. stranger) x 2 (future reinforcement: CS+ vs. CS-) repeated-measures ANOVA examining averaged responding across all 4 trials of the habituation stage for each CS+ and CS- in each condition revealed that there was no interaction of condition and future reinforcement, F(1,30)=.292, p=.593, η_p_^2^=.010, and no main effect of future reinforcement, F(1,30)=.211, p=.649, η_p_^2^=.007, although there as a main effect of condition such that SCR for social support figure images was higher than that for stranger images, F(1,30)=5.068, p=.032, η_p_^2^=.145. This main effect of condition is expected given the emotionally arousing nature of the relationship with a social support figure. Further examination using paired-samples t-tests revealed that SCR for the future CS- did not significantly differ from that for the future CS+ in either the social support, t(30)=.602, p=.552, 95% CI [-0.04, 0.07], or stranger condition, t(30)=-.182, p=.875, 95% CI [-0.04, 0.03]. Thus, no pre-existing characteristics leading to differing SCR for the future CS+ or CS- in either condition could account for later differences in SCR after the learning procedures.

**Conditional Responding from Acquisition to Extinction**

Given that there was no conditional fear response present in the social support condition at the end of the fear acquisition stage, it is difficult to interpret any change from the end of acquisition to the beginning of extinction with any certainty. However, evaluation of whether there was any difference in change in conditional responding from the end of acquisition to the beginning of extinction across the social support and stranger conditions may provide further insight into the nature of these responses, therefore we present this information here. A repeated-measures 2 (stage: end of acquisition (final 5 trials), beginning of extinction (first 2 trials)) x 2 (condition: social support, stranger) x 2 (reinforcement: CS+, CS-) ANOVA revealed no significant 3-way interaction, F(1,30)=.067, p=.797, η_p_^2^=.002, indicating there was no difference in changes in conditional responding for the CS+ and CS- in the social support or stranger condition from the end of acquisition to extinction. Further assessment via paired-samples t-tests comparing difference scores (CS+ - CS-) revealed that there was no significant change in SCR from the final 5 trials of acquisition to the first two trials of extinction in each condition in either the social support condition, t(30)=1.034, p=.304, 95% CI [-0.04,0.13], or the stranger condition, t(30)=1.102, p=.279, 95% CI [-.05,.16]. Thus, the trajectory of conditional responding from the end of fear acquisition to the beginning of fear extinction did not differ across conditions.

**Participant-wise SCR**

Figures S1-S3 display the difference scores (SCR for CS+ - SCR for CS-) for each participant (n=31), in each condition (social support, stranger) for the stages of interest: first five trials of the acquisition stage (Figure S1), the second 5 trials of the acquisition stage (Figure S2), and the first two trials of the extinction stage (Figure S3).

**Trial-by-trial SCR**

Figures S4 and S5 show the trial-by-trial skin-conductance-responses for each CS+ and CS- across the acquisition (Figure S4) and extinction (Figure S5) phases.

*References*

Bradford, D. E., Starr, M. J., Shackman, A. J., & Curtin, J. J. (2015). Empirically based comparisons of the reliability and validity of common quantification approaches for eyeblink startle potentiation in humans. *Psychophysiology*, *52*(12), 1669-1681.

Hornstein, E. A., & Eisenberger, N. I. (2017). Unpacking the buffering effect of social support figures: social support attenuates fear acquisition. *PloS one*, *12*(5).

Hornstein, E. A., Fanselow, M. S., & Eisenberger, N. I. (2016). A safe haven: Investigating social-support figures as prepared safety stimuli. *Psychological science*, *27*(8), 1051-1060.

Hornstein, E. A., Haltom, K. E., Shirole, K., & Eisenberger, N. I. (2018). A unique safety signal: Social-support figures enhance rather than protect from fear extinction. *Clinical psychological science*, *6*(3), 407-415.

Figner, B., & Murphy, R. O. (2011). Using skin conductance in judgment and decision making research. In M. Schulte-Mecklenbeck, A. Kuehberger, & R. Ranyard (Eds.), *A handbook of process tracing methods for decision research* (pp. 163-184). New York, NY: Psychology Press.

Lonsdorf, T. B., Menz, M. M., Andreatta, M., Fullana, M. A., Golkar, A., Haaker, J., Heitland, I., Hermann, A., Kuhn, M., Kruse, O., Drexler, S. M., Meulders, A., Nees, F., Pittig, A., Richter, J., Romer, S., Shiban, Y., Schmitz, A., Straube, B., Vervliet, B., Wendt, J., Baas, J. M. P., & Merz, C. J. (2017). Don’t fear ‘fear conditioning’: Methodological considerations for the design and analysis of studies on human fear acquisition, extinction, and return of fear. *Neuroscience & Biobehavioral Reviews*, *77*, 247-285.

Northoff, G., Heinzel, A., De Greck, M., Bermpohl, F., Dobrowolny, H., & Panksepp, J. (2006). Self-referential processing in our brain—a meta-analysis of imaging studies on the self. *Neuroimage*, *31*(1), 440-457.

Olsson, A., Ebert, J. P., Banaji, M. R., & Phelps, E. A. (2005). The role of social groups in the persistence of learned fear. *Science*, *309*(5735), 785-787.

Phelps, E. A., Delgado, M. R., Nearing, K. I., & LeDoux, J. E. (2004). Extinction learning in humans: role of the amygdala and vmPFC. *Neuron*, *43*(6), 897-905.

Schiller, D., Monfils, M. H., Raio, C. M., Johnson, D. C., LeDoux, J. E., & Phelps, E. A. (2010). Preventing the return of fear in humans using reconsolidation update mechanisms. *Nature*, *463*(7277), 49-53.

Tzourio-Mazoyer, N., Landeau, B., Papathanassiou, D., Crivello, F., Etard, O., Delcroix, N., ... & Joliot, M. (2002). Automated anatomical labeling of activations in SPM using a macroscopic anatomical parcellation of the MNI MRI single-subject brain. *Neuroimage*, *15*(1), 273-289.
